# Linking mixing processes and climate variability to the heat content distribution of the Eastern Mediterranean abyss

**DOI:** 10.1038/s41598-018-29343-4

**Published:** 2018-07-27

**Authors:** Vincenzo Artale, Federico Falcini, Salvatore Marullo, Manuel Bensi, Florian Kokoszka, Daniele Iudicone, Angelo Rubino

**Affiliations:** 1 Agenzia Nazionale per le Nuove Tecnologie, l’Energia e lo Sviluppo Economico Sostenibile, ENEA — Centro Ricerche Frascati - Dipartimento Sostenibilità dei Sistemi Produttivi e Territoriali (SSPT), Frascati, 00044 Italy; 2 Istituto di Scienze dell’Atmosfera e del Clima, Consiglio Nazionale delle Ricerche, Roma, 00133 Italy; 3 Istituto Nazionale di Oceanografia e di Geofisica Sperimentale, OGS, Trieste, 34010 Italy; 4 Stazione Zoologica Anton Dohrn, Villa Comunale, Napoli, 80121 Italy; 5 Department of Environmental Sciences, Informatics and Statistics, University of Venice, Mestre, 30172 Italy

## Abstract

The heat contained in the ocean (OHC) dominates the Earth’s energy budget and hence represents a fundamental parameter for understanding climate changes. However, paucity of observational data hampers our knowledge on OHC variability, particularly in abyssal areas. Here, we analyze water characteristics, observed during the last three decades in the abyssal Ionian Sea (Eastern Mediterranean), where two competing convective sources of bottom water exist. We find a heat storage of ~1.6 W/m^2^ – twice that assessed globally in the same period – exceptionally well-spread throughout the local abyssal layers. Such an OHC accumulation stems from progressive warming and salinification of the Eastern Mediterranean, producing warmer near-bottom waters. We analyze a new process that involves convectively-generated waters reaching the abyss as well as the triggering of a diapycnal mixing due to rough bathymetry, which brings to a warming and thickening of the bottom layer, also influencing water-column potential vorticity. This may affect the prevailing circulation, altering the local cyclonic/anticyclonic long-term variability and hence precondition future water-masses formation and the redistribution of heat along the entire water-column.

## Introduction

Convection and diapycnal mixing contribute to transfer and redistribute water masses and heat throughout the deep ocean^1–3^. These phenomena act at very different time scales^4,5^. Diapycnal mixing, in particular, increases the potential energy within a stratified fluid by raising the water mass center on a larger time and spatial scale. It is triggered by an external process^4,6^ and it is concentrated above seamounts, mid-ocean ridges, and along strong currents^2,3^.

Despite its thorough implications in the ocean circulation, the relationship between the intensity of overturning circulation and deep mixing rates is not yet fully understood, particularly, in the Mediterranean Sea^7–11^. Numerical models, in such a context, seems often useless since they are too sensitive to vertical eddy diffusivity and largely affected by inaccuracy at deep layers^12–14^. Consequently, the analysis of *in situ* observations is crucial for understanding the actual role of mixing in the deep ocean circulation and heat content distribution.

**Figure 1:**
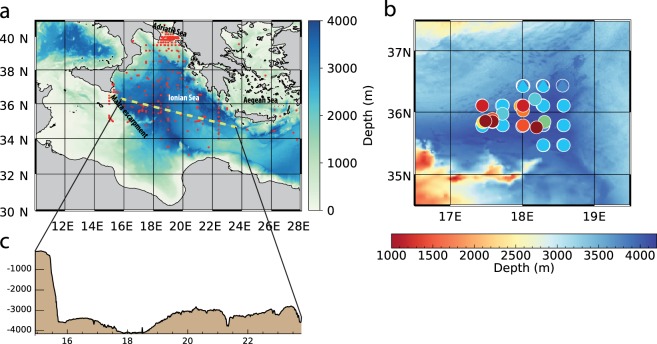
The study area. (**a**) Bathymetry of the Ionian Sea. The red dots indicate the CTD casts we consider in this work (collected from 1977 to 2011); the yellow line indicates the bathymetric section of panel (c). (**b**) Zoom-in of the study area with the deep CTD casts (those reaching 4000 m depth); colors correspond to the temperature and salinity profiles in Figs 2a,b and 4a. Maps are generated by IDL 8.0 (www.harrisgeospatial.com/IntelliEarthSolutions/GeospatialProducts/IDL.aspx).

**Figure 2:**
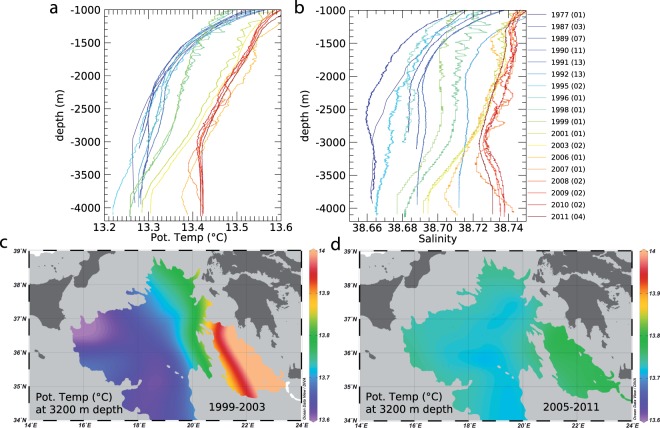
Hydrographic results. (**a**) Temperature profiles. (**b**) Salinity profiles; colors indicate the year of data collection, as also reported in Fig. 4a while in parenthesis we report the number of stations that were averaged in the same year. (**c**) Horizontal temperature map at 3200 m depth, as obtained from CTD measurements collected from 1999 to 2003. (**d**) Horizontal temperature map at 3200 m depth, as obtained from CTD measurements collected from 2005 to 2011. Figure created using Ocean Data View software (ODV - version, 4.7.4., Schlitzer, R., Ocean Data View, odv.awi.de, 2017).

The Eastern Mediterranean Transient (EMT), i.e., the first experimental evidence of a non-steady behavior of the deep Mediterranean thermohaline circulation, gave us the opportunity to investigate, experimentally, convective and mixing dynamics^7^. During the EMT (occurred between the end of 80′ and the beginning of 90′), the Aegean Sea turned to be the source of deep water, also causing an increase of surface water temperature, salinity, and density in the Eastern Mediterranean and, in particular, in the Aegean Sea^7,9^ (Fig. 1). This dense water feeds the Eastern Mediterranean Deep Water (EMDW), thus replacing (for some years) the Adriatic Sea (Fig. 1) as the main producer of bottom water^7,9,15^. Subsequently, the meridional overturning circulation of the Eastern Mediterranean, as obtained by general circulation models, showed multiple equilibria states^16^ under slight perturbations of the present-day-like conditions^17,18^. These findings revealed two stable states and a hysteresis behavior of deep-water formation in the Adriatic Sea, when the atmospheric (restoring) temperature over the Aegean Sea is tuned^18^. A similar hysteresis could also affect the abyssal Ionian long-term variability. The Ionian abyssal layer (from 3000 to 4000 m depth) is indeed undergoing a warming and salinification phase, started after the EMT and likely associated with an active Mediterranean overturning circulation state^18^. An interplay between advection and mixing processes may be therefore at the base of the anomalous heat storages that characterized the Eastern Mediterranean basin in the last three decades^19^ (Fig. 2 and Supplementary Fig. S1).

The Ionian abyssal plain (Fig. 1) is characterized by complex topographic boundaries (Fig. 1b). In the western sector of this plain, the EMDW, which results from the mixing of dense waters coming from the Adriatic and Aegean basins, is constrained to flow along the very steep Malta Escarpment (topographic gradient up to 30°), with a large vertical displacement of the seafloor, i.e., about 3000 m (Fig. 1a,b). In the eastern sector, the same EMDW that accumulated in the abyssal Ionian flows over a very irregular bathymetry, between 3000–4000 m depth, and it is also constrained by the Mediterranean Ridge, which raises till 2000 m depth. This results in the activation of mixing processes that are triggered by deep waters flowing over uneven bathymetry, as mixing is much higher in topographic troughs or canyons than on ridges/crests^2^. We present a thorough hydrographic analysis that shows how all this is at the base of a pseudo-autogenic variability of the Ionian abyssal plain circulation and its heat content, where the seafloor-induced, diapycnal mixing plays a crucial role in warming and thickening the bottom layer.

## Advection, Mixing, and Warming Processes in The Lonian Abyss

We focus here on the interaction between mixing and the alternate advection of abyssal waters that are produced by two different sub-basins: the Adriatic and Aegean Seas^20^. Conductivity-Temperature-Depth (CTD) casts in the Ionian Sea (Figs 1 and 2) show significant changes in the deep thermohaline structure, giving indications on internal exchange mechanisms (Fig. 2). Data range from the pre-EMT to the present-day state, covering a period of more than 30 years (from 1977 to 2011), and show two distinct states (Fig. 2). An “original” state (i.e., 1977; Fig. 2a,b) was characterized by a relatively fresh (38.66), cold (13.26 °C), homogeneous bottom layer between 3000 and 4000 m depth. This state was then perturbed by the EMT (occurred during the 90 s in this region), which introduced a saltier and warmer water of Aegean origin, making the deep-water column well stratified (Fig. 2a,b). This well-stratified condition progressively changed towards a “new” homogeneous state at the bottom layer, observed from 2003 to 2011 (yellowish profiles in Fig. 2a). During this last phase, the data show a warming process of the bottom layer that consequently brought to the formation of a 1000-m-thick, relatively warm (~13.42 °C) and salty (~38.73), homogeneous layer (see reddish profiles in Fig. 2a,b). This brings to the intriguing question: did this heat content anomaly (ΔT ~ 0.2 °C) at 4000 m depth - and thus the thickening/homogenizing process described here - come from vertical^21,22^ and/or lateral^10,15,23–25^ mixing processes? We therefore envision that the “new” bottom Ionian water results from the continuous entrainment of the warmer, upper waters, a process that would cause a loss of kinetic energy and gain of potential energy in the deep layer^4^. Such phenomena would explain the link between mixing processes and the heat content redistribution within the abyssal Ionian, also accounting for the observed long-term variability in response to the climate changes that the whole Mediterranean Sea is undergoing in the last decades^11,15,18,26^. Indeed, stratification of the abyssal part of the Ionian Sea reflects the buoyancy flux variability from the Adriatic Sea^27^. This advective process is found at work also in numerical simulations^18^: cold events (similar to the EMT) would produce a buoyancy transport at the source-water site of the Eastern Mediterranean, also triggering a hysteresis behavior due to the intrinsic non-linearity of the Adriatic-Ionian-Aegean system.

**Figure 3:**
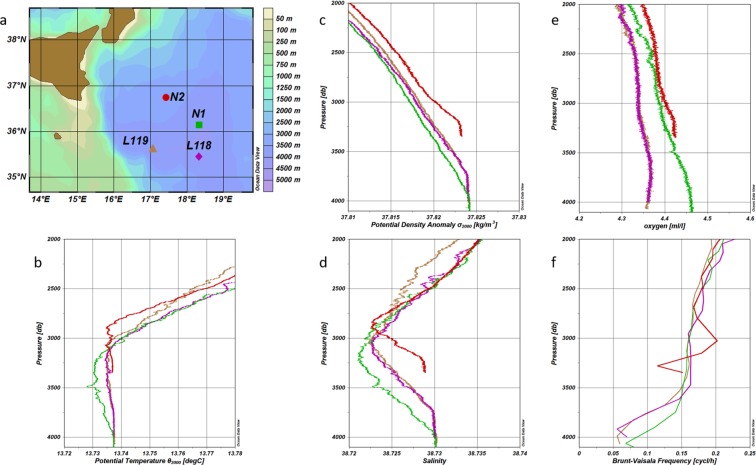
Hydrographic characteristics of the 2011 dataset. (**a**) Geographic location of the stations N1, N2, L118, and L119. (**b**) Potential temperature profiles for the four stations. (**c**) Potential density anomaly profiles for the four stations. (**d**) Salinity profiles for the four stations. (**e**) Oxygen profiles for the four stations. (**f**) Brunt–Väisälä frequency profiles for the four stations. Colours refer to the stations in panel (**a**). Figure created using Ocean Data View software (ODV - version, 4.7.4., Schlitzer, R., Ocean Data View, odv.awi.de, 2017).

To analyze, at local scale, the crucial role of mixing in the Ionian abyss we consider a case study, taking into account four synoptic CTD casts, collected in the 2011 (Fig. 3). Density profiles at stations L118, L119, and N1 (Fig. 3) are similar to those theoretical profiles that mark a loss of kinetic energy occurring between the onset of turbulence in the stratified mixing layer and its decay (Fig. S2), while the potential energy of the mean stratification increases^21^. On the other hand, the density profile at N2, from 3000 m depth, is more stratified. Moreover, both steepness and thickness of the bottom homogeneous layer in this station are less enhanced than those observed in the other southern stations (Fig. 3). All this marks, at this station, a preconditioning phase for Kelvin–Helmholtz instability (i.e., precondition and willingness for mixing), as confirmed by the Brunt–Väisälä frequency profiles^21^ (Fig. 3e,f). By tracking these mixing processes we can argue, therefore, that the CTD profile at the N2 station (i.e., no far from the deep convection area of the Southern Adriatic) represents an incoming scenario, driven by advection of dense Adriatic water in which turbulence is still not fully developed. Mixing, likely induced by bottom roughness, steepness, and shear instabilities^21,28^, would bring the more oxygenated, well-stratified upstream water column (sampled in N2) to a thick, homogeneous bottom layer, as observed at stations L118, L119, and N1 (Fig. 3 and Supplementary Figs S2 and S3). This is justified by the fact that the “stratified tongue” of Adriatic origin, by reaching intermediate depths and carrying near-surface ocean properties, interacts – southward – with the steep topography of the Malta escarpment and with the upper fluid. South, deep CTD stations show a smaller Brunt–Väisälä frequency, which reveals a reduced stability in the deep part of the water column that favours internal mixing (Fig. 3e,f and Supplementary Figs S2 and S3).

The complexity of the Eastern Mediterranean topography forces the AdDW to flow along a large vertical displacement (about 3000 m) and on a very irregular seafloor, with a significant velocity magnitude of the water masses^10^. Due to lateral shearing, horizontal diffusion strongly affects the temporal evolution of the deep hydrographic characteristics of the basin. Potential temperature and salinity patterns at 3200 m depth, during the period 1999–2003, showed a well-defined horizontal gradient that confirms the precondition to mixing processes, viz., the strong interaction between two distinct dense water masses (Fig. 2c,d). These patterns were indeed totally smoothed during the phase 2005–2011, when the well-stratified (both vertical and horizontal) condition was no longer observed (Fig. 2d). The spreading of these two water masses from different sources (i.e., Adriatic and Aegean) was likely enhanced by their interaction with the bathymetry^29^.

**Figure 4:**
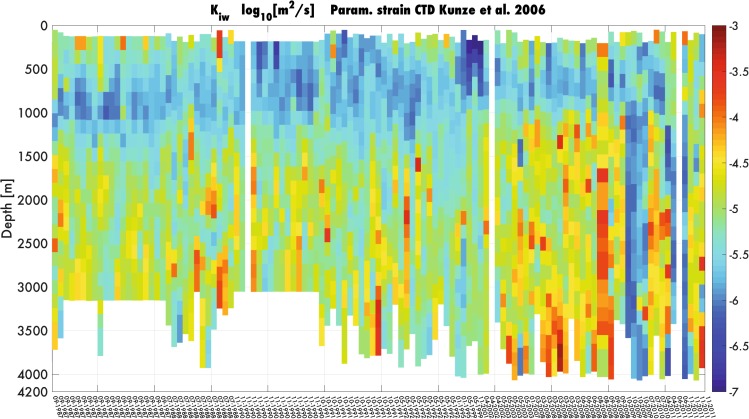
Hovmöller diagram of the kinetic turbulent diffusion rate (K_IW_). The diffusion rate is calculated from equation (1), by using the Osborn-Cox relation^32^. We use here vertical segments of 320 m. Segments are defined along the vertical grid from the bottom to 50 m from the surface to avoid contamination by surface processes. Signal is de-trended and a Tukey windowing is applied. Variance loss is corrected by multiplying by a factor 1.07. The Hovmöller diagram shows an increase of bottom-induced kinetic turbulent diffusion rate from the onset of the 2003, where the post-EMT baroclinc structure appears in the bottom layer (see Fig. 5b).

To explore topographic-induced diapycnal mixing we calculate dissipation rates from the “CTD strain-based” parameterization^30^ (Fig. 4). In particular, we use CTD profiles to determine the isopycnal vertical strain, by assuming that a part of the variance is due to the presence of internal waves (IWs). This variance estimates local energy of the IW field that modulates the canonical value of kinetic turbulent dissipation rates ${\varepsilon }_{0}$, which is given by the Garrett and Munk’s model (GM)^30–32^:

1 $${\varepsilon }_{IW}={\varepsilon }_{0}{(\frac{N}{{N}_{0}})}^{2}\frac{{\langle {\xi }_{z}^{2}\rangle }^{2}}{{\langle {\xi }_{z}^{2}\rangle }_{GM}^{2}}{F}_{2}({R}_{\omega })\lambda (f,N),$$

where $\langle {{\rm{\xi }}}_{{\rm{z}}}^{2}\rangle$ is the isopycnal strain variance, ${{\rm{F}}}_{2}({{\rm{R}}}_{{\rm{\omega }}})=\,\frac{{{\rm{R}}}_{{\rm{\omega }}}({{\rm{R}}}_{{\rm{\omega }}}+1)}{6\sqrt{2}\sqrt{{{\rm{R}}}_{{\rm{\omega }}}-1}}$ is the frequency content of the internal wave’s packet^30^, ${{\rm{R}}}_{{\rm{\omega }}}=3$ is the strain rate, and ${\rm{\lambda }}({\rm{f}},{\rm{N}})=\frac{{f\cosh }^{-1}({\rm{N}}/{\rm{f}})}{{{\rm{f}}}_{0}{\cosh }^{-1}({{\rm{N}}}_{0}/{{\rm{f}}}_{0})}$ represents the latitude effect; here ε_0_ = 7 × 10^−10^ W/kg, ${{\rm{f}}}_{0}=5.2\times {10}^{-3}\,\mathrm{rad}/{\rm{s}}$, and ${{\rm{N}}}_{0}\equiv 3\,\mathrm{cph}$^33,34^. In (1), the ispocynal strain ${{\rm{\xi }}}_{{\rm{z}}}=\frac{{{\rm{N}}}^{2}-{{\rm{N}}}_{{\rm{z}}}^{2}}{{\bar{{\rm{N}}}}^{2}}$ is calculated by considering the mean value of the Brunt-Vaisala frequency along the vertical segment of the water column (${\bar{{\rm{N}}}}^{2}$) and the IW density perturbation (${{\rm{N}}}^{2}-{{\rm{N}}}_{\langle z\rangle }^{2}$). From ${{\rm{\varepsilon }}}_{{\rm{IW}}}$ we then estimate the kinetic turbulent diffusion rate ${{\rm{K}}}_{{\rm{IW}}}={{\rm{\Gamma }}{\rm{\varepsilon }}}_{{\rm{IW}}}{{\rm{N}}}^{-2}$^35^. Our analysis provides a quantitative estimation of diapycnal mixing due to the rough bathymetry, i.e., ${{\rm{K}}}_{{\rm{IW}}}$ ~ 10^−4^ m^2^/s, and clearly shows the role of sea bottom in enhancing isopycnal vertical strain, which occurred from the onset of the EMT (Fig. 4).

## Baroclinic Vertical Modes and Equilibrium States

To investigate fluctuations of potential temperature and salinity, observed from CTD vertical profiles (Figs 2 and 3), we decomposed the deep subset of hydrographic data into vertical modes, i.e., the oscillatory signals of those few homogeneous-like layers (i.e., equivalent depths *h*_*n*_) that well represent the density stratification within the water column^36^.

External forcing (typically wind stress), or dense water injections at intermediate or deep layers, affects ocean (as well as the Mediterranean basin) stratification, causing oscillatory signals that strongly depend on “equivalent depths”^36^. By assuming a sinusoidal water pressure, oscillatory signals can be indeed separated from the equation of motion to yield discrete *n* vertical shapes of variability that, in turn, define the separation constants $g{h}_{n}=\frac{1}{({k}^{2}+{l}^{2})}$ and the resulting horizontal scales (i.e., the internal Rossby radius) ${L}_{R,n}=\frac{N{h}_{n}}{n\pi f}$, where *N* is the buoyancy frequency and *f* the Coriolis parameter^36^.

Propagation of Rossby waves is governed by the quasi-geostrophic potential vorticity equation^37^:

2 $$\frac{\partial }{\partial t}[{{\nabla }}^{2}p+\rho \frac{\partial }{\partial z}(\frac{1}{\rho {N}^{2}}\frac{\partial p}{\partial z})]+\beta \frac{\partial p}{\partial x}=0,$$

where p is pressure, *ρ* is density, *N* is the buoyancy frequency and *β* is the latitudinal variation of planetary vorticity. By assuming a solution (i.e., the sinusoidal water pressure) of the $p(x,\,y,\,z,\,t)=p(z){e}^{i(kx+ly-\omega t)}$, equation (2) becomes:

3 $$\rho \frac{\partial }{\partial z}(\frac{1}{\rho {N}^{2}}\frac{\partial p}{\partial z})+\frac{1}{g{h}_{n}}p=0,$$

where $g{h}_{n}=\frac{1}{(\frac{\beta \kappa }{\omega }+{k}^{2}+{l}^{2})}$ is the separation constant and *g* the gravity. Equation (3) can be solved with the aid of potential temperature and salinity profiles (from CTD casts), by considering the following boundary conditions (respectively at the top and bottom of a layer *H*):

4 $$\frac{\partial p}{\partial z}+\frac{{N}^{2}}{g}p=0,\,\,z=0\,{\rm{and}}\,\frac{\partial p}{\partial z}=0,\,\,z=-\,H.$$

The resulting horizontal scales (*L*_*R,n*_) are therefore obtained as ${L}_{R,n}=\frac{N{h}_{n}}{n\pi f}$. By using boundary condition (4) at the sea surface and a rigid boundary condition at the bottom, the barotropic mode is ill-determined. However, all solutions remain acceptable, because of the intrinsic depth independence of the barotropic mode.

We obtain *n* vertical shapes of variability that show different ranges of scale of motion as well as strong inter-annual (decadal) variability in the stratification, which requires a linear combination of many baroclinic modes (Supplementary Fig. S4). Based on zero-crossings of the baroclinic modes, and thus the associated *h*_*n*_, we computed the resulting internal Rossby *radius* (*L*_*R,n*_), which varies between 5–30 km and 50–150 km. This two-range behavior implies a persistent exchange of energy between the typical scales of motion^38^.

**Figure 5:**
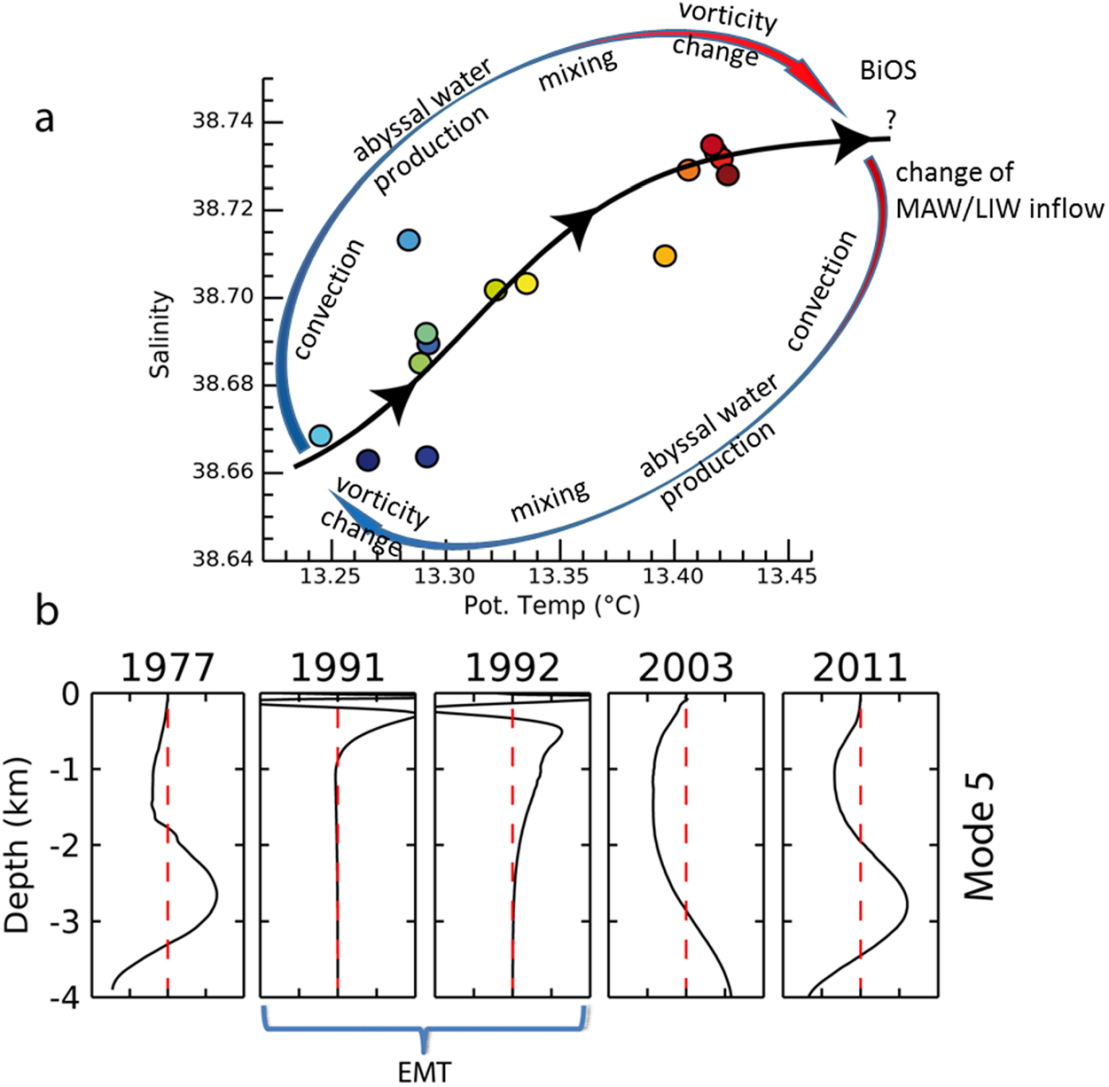
Equilibrium states and baroclinic modes. (**a**) T-S diagram highlighting the two different states of the deep Ionian Sea (i.e., fresh and cold during the period 1977–1999, salty and warm from the 2003). The loop qualitatively schematizes all the processes that would be involved in the hypothesized hysteresis cycle: convection brings to abyssal water production; mixing processes due to bathymetric constraints bring then to the decay of vorticity (see Fig. 6b) in the bottom layer that would bring to Adriatic-Ionian Bimodal Oscillating System (BiOS) [ref.^5^]; this, in turn, regulates the inflow of Modified Atlantic and Levantine Intermediate waters (MAW and LIW, respectively). (**b**) The 5^th^ baroclinic mode as obtained from the CTD casts (see text); starting from the 1977, where two equivalent depths are observed at 2000 and 3200 m depth, the panel shows that the EMT stretched baroclinic structures upward, removing the baroclinicity in the deep layers; the onset of a baroclinc structure in the bottom layer appears again from 2003 (see Fig. 4) while the 2011 shows similar equivalent depths of those observed in 1977. Figure created using IDL 8.0 (www.harrisgeospatial.com/IntelliEarthSolutions/GeospatialProducts/ IDL.aspx).

To account for deep, less energetic (in respect to the leading modes) variability, we focus on the 5^th^ mode. This mode allows us to analyze the baroclinic structure between 1000 and 4000 meters, since the first four baroclinic modes are not able to fully capture any equivalent depths within this range (Supplementary Fig. S4). Indeed, the 5^th^ mode shows two equivalent depths in the initial state (i.e., 1977) at 2000 and 3200 m depth, respectively (Fig. 5b). These likely represent an “equilibrium state” in terms of density stratification, where the deepest equivalent depth (i.e., from ~3200 m depth to the bottom) marks the presence of Adriatic dense water. During the EMT, the intrusion of Aegean water induced a new baroclinic state (Fig. 5b) due to a strong stratification from the bottom up to the sub-surface layers, which uplifted the deepest equivalent depth till 1000 m depth, by stretching the whole abyssal layer^39^ and increasing the internal Rossby radius till ~150 km. In this quasi-barotropic state, local mixing in the deep layers was negligible. In 2003 (i.e., more than 10 years later, see also Fig. 5b), the 5^th^ mode shows the re-activation of the deep equivalent depth at 3000 m depth. CTD casts (Fig. 2) show that this was likely due to the work made by different sources of mixing (i.e., bottom roughness, shearing effects, and topographic constraints)^23^ that acts in absence of extreme external forcing (Supplementary Figs S2 and S3), like those occurring during the EMT^40^. As a consequence of the re-activation of the deep equivalent depth, observed in the 2003, the 2011 showed a baroclinic pattern similar to the one observed in the 1977 (Figs 2a and 5b); this brings us to argue the presence of new “equilibrium state”. Indeed, although characterized by a deep layer, warmer and saltier than the one in 1977, the 2011 shows identical characteristics in terms of stratification of the original equilibrium state (i.e., 1977). In case of an eventual comeback to an initial state, we argue a similar convection-mixing process that may not necessarily pass through a different hydrographic path. Indeed, it is reasonable to assume that the strong nonlinearity of mixing processes would favour a hysteresis cycle^17,18^ (Fig. 5a).

## Heat content redistribution and potential vorticity diagnosis

From CTD casts we estimate an OHC variation in the deep layer ${\rm{\Delta }}Q\cong 0.14\times {10}^{21}J$ (see Materials and Methods), which is equivalent to 1.62 W/m^2^. This flux is more than double of the global mean anomaly, more likely due to the climate change^41–43^: the deep layer in the Ionian basin is a non-negligible reservoir of heat. We envision such an OHC variation and, in particular, what we observe from 2003 as a sink process of potential vorticity (Π), where bottom drag and entrainment are mutual ways to exchange momentum and heat within the subsurface layer. We thus pursue a diagnostic approach by considering the following Ertel theorem for potential vorticity evolution^44^:

5 $$\frac{d{\rm{\Pi }}}{dt}=\frac{\nabla T}{\rho }\cdot (\nabla \times \frac{ {\mathcal F} }{\rho }),$$

where potential vorticity is defined as ${\rm{\Pi }}\equiv \frac{(\overrightarrow{\omega }+2\overrightarrow{{\rm{\Omega }}})}{\rho }\cdot \nabla T$, with $\overrightarrow{\omega }$ and $2\overrightarrow{{\rm{\Omega }}}$ the relative and planetary vorticity, respectively, and *ρ* the water density. In Eq. (5) we assume a quasi-barotropic flow, i.e. a flow for which the water density can be assumed to be function of the temperature [$\rho (T,S) \sim \rho (T)$)]; this allows us to neglect baroclinic effect that might dissipate potential vorticity^44^. Moreover, by assuming adiabatic conditions we can also disregard potential vorticity dissipation due to non-adiabatic effects^44^. Finally, in equation (5) ${\mathcal F} =K|\overrightarrow{u}|\overrightarrow{u}$ represents the frictional force, not negligible for our case, encompassing friction due to both bottom drag and entrainment with the upper layer^45–48^, where *K* (m^−1^) and $\overrightarrow{u}$ (ms^−1^) are the frictional coefficient and a bulk velocity of the bottom current.

By considering the sole vertical contribution of the three vorticity flow components (i.e., ${\zeta }\equiv \frac{\partial v}{\partial x}-\frac{\partial u}{\partial y}$), equation (5) can be approximated as

6 $$\frac{d{\rm{\Pi }}}{dt}=-\frac{K}{\rho }|\langle \overrightarrow{u}\rangle |\zeta \frac{\partial T}{\partial z},$$

*ζ* (s^−1^) is the relative vorticity of the flow and $\frac{\partial T}{\partial z}$ the temperature vertical gradient of the water column.

From the definition of Π (and after some cumbersome algebra), equation (6) gives the following vorticity equation:

7 $$\frac{d\zeta }{dt}+(\zeta +f)\frac{d}{dt}ln\frac{\partial T}{\partial z}=-\,\frac{K}{\rho }\langle |\overrightarrow{u}|\rangle \zeta .$$

**Figure 6:**
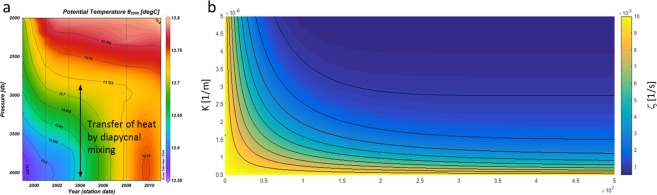
PV stretching and change of vorticity. (**a**) Hovmöller diagram of the temperature profiles at the deepest layer from 1999 to 2011; from the onset of mixing processes the OHC variation in the deep layer is ~*0.14* × 10^21^ J. (**b**) Decay of vorticity due to mixing and friction as a function of time and the frictional coefficient *K* (m^−1^).

Equation (7) can be used to diagnose how mixing processes that led to changes in temperature stratification ($\frac{d}{dt}ln\frac{\partial T}{\partial z}$; Fig. 2a) affect the whole circulation of the bottom layer. Analytic solution for ζ shows a significant decay of vorticity, mechanistically describing the intrinsic relation among friction, the induced mixing (Figs 2 and 6), and the loss of circulation (i.e., the vorticity) in the Ionian Sea, during the last decade^7,10^. The onset of the EMT and thus the triggering of bottom-induced mixing processes would therefore cause a thermal stretching of the water column (Fig. 6). In such a context, the potential vorticity model shows that kinetic energy of the mean circulation is lost with time while the potential energy of the mean stratification increases^21^. Consequently, the bottom-up, friction-driven loss of vorticity would bring to a change of the whole baroclinic structure of the basin through upwelling (Figs 5b and 6b). All this represents an additional contribution to the Stommel-Aron theory, by highlighting the role of friction-induced mixing processes that act at basin scale that more significantly contribute to the local circulation of whole water column^22,49^.

## Conclusions

Our analysis reveals that coexisting stable stratification, deep mixing, intense meridional overturning circulation, and mesoscale eddies affect the heat content redistribution within the Eastern Mediterranean abyss. These features raise fundamental questions concerning the ocean circulation energetics and, on the other hand, prove that small-scale mixing processes are necessary to resupply the potential energy that is removed in the interior by the overturning, upwelling, and eddy-generating process^16,28,50^.

These mixing processes, by acting as a sink of potential vorticity, give rise to decadal variation of vorticity that, in turn, may lead to interchanges of cyclonic/anti-cyclonic phases in the upper ocean^9^, depending on the baroclinic structure of the entire water column. Adriatic inflow of intermediate and surface waters (i.e., the Levantine Intermediate Water and the Modified Atlantic Water), and thus the Adriatic pre-conditioning convection, would be dramatically affected by the bottom-up, mixing-driven changes of polarity (i.e., cyclonic vs. anti-cyclonic behavior) of the surface circulation.

At global scale, our findings give support to what recently outlined by several authors (e.g., Ferrari *et al*.^51^; McDougall and Ferrari^52^; Shu *et al*.^22^) in resolving the conundrum of how bottom waters are transformed into lighter waters. Indeed, estimates of the zonally averaged global overturning circulation, based on both inverse calculations from ocean observations^53,54^ and numerical models constrained to observations^55^, showed that bottom waters slowly rise throughout the rest of the oceans crossing density surfaces at least up to 2000 m^56^. Moreover, there is growing evidence from *in situ* measurements that the turbulent kinetic energy generated by breaking internal waves is large within a few hundred meters of rough-bottom topography and decays to weaker values farther up in the water column. The bottom enhancement of turbulence reflects the generation of energetic waves, impinging over topography and breaking locally^22,28,57^.

## Materials and Methods

CTD data are from the ICES Dataset on Ocean Hydrography (The International Council for the Exploration of the Sea, Copenhagen, 2014), the HNODC-Hellenic National Oceanographic Data Centre, and from the SeaDataNet (pan-European network for oceanographic and marine data and information management), and the MEDAR/MEDATLAS project (Supplementary Information).

CTD profiles show Potential Temperature referenced to the sea surface (0 decibars).

Analytic solution of equation (6) is obtained by considering the following first-order Ordinary Differential Equation: $\frac{d\zeta }{dt}+A(t)\zeta +B(t)=0$, where $A(t)=\frac{K}{\rho }\langle |\overrightarrow{u}|\rangle +\frac{d}{dt}ln\frac{\partial T}{\partial z}$ and $B(t)=f\frac{d}{dt}ln\frac{\partial T}{\partial z}$. We estimate a variation of the temperature stratification from ~10^−4^ °C/m to 0 °C/m in ~10 years (Fig. 2); for sake of simplicity, the bulk velocity $|\overrightarrow{u}|$ is taken as a constant ~0.05 m/s^22^.

According to Fig. 2a, OHC variation was estimated by assuming a temperature increase of *δT* ~ 0.2 °C within a water thickness *δZ* ~ 1000 m, and deep surface A ~70000 km^2^ at ~3200 m depth: ${\rm{\Delta }}Q=\rho {C}_{p}\,\delta T\delta Z\,A\cong 0.14\times {10}^{21}J$, which is equivalent to 1.62 W/m^2^.

For the kinetic turbulent diffusion rate (K_IW_), the variance is determined by integrating vertical spectrum over chosen band-width that we assume representing the internal waves vertical wavelengths. We work here with 320 m vertical segments, that is, we can solve 320 to 2 m wavelengths for strain (because of the limit due to the Nyquist’s theorem depending on vertical resolutions, i.e. 1 m for CTD casts considered in this work). Segments are defined along the vertical grid from the bottom to 50 m from the surface to avoid contamination by surface processes. A residue of the water column division by the segment length can remain, in consequence of what a non-processed part can exist at the top of certain profiles. Signal is de-trended, and a Tukey windowing is applied. Variance loss is corrected by multiplying by afactor 1.07. Integration [320–30 m] follows the recommendation proposed by Kunze *et al*.^27^. We implemented the criteria proposed by Gargett^54^, that takes account of the possible energy’s saturation of the local internal wave field. Each segment is integrated from 320 m to a locally variable cut-off vertical wavelength/wavenumber Ultimately limited to the lower bounds, defined previously (30 m for strain). Integration range is here I_strain_ = [320 m to 30 m]. Then we overlap segments to produce estimations on a 160 m-vertical grid.

## Electronic supplementary material

Supplementary Information
