# Supplementary material for: Linking mixing processes and climate variability to the heat content distribution of the Eastern Mediterranean abyss
Source: Sci Rep. 2018 Jul 27;8:11317. doi: 10.1038/s41598-018-29343-4 (PMC6063911; doi:10.1038/s41598-018-29343-4)
Supplement: Supplementary file 1 — Supplementary Information [file 41598_2018_29343_MOESM1_ESM.pdf]

Supplementary Information

for

Linking mixing processes and climate variability to the heat content distribution of the Eastern Mediterranean abyss

Vincenzo Artale[1,2], Federico Falcini[2*], Salvatore Marullo[1,2], Manuel Bensi[3], Florian Kokoszka[4], Daniele Iudicone[4], Angelo Rubino[5]

[1] *Agenzia Nazionale per le Nuove Tecnologie, l'Energia e lo Sviluppo Economico Sostenibile, ENEA — Centro* Ricerche *Frascati - Department for Sustainability (SSPT), Frascati 00044, Italy*

[2] *Istituto di Scienze dell'Atmosfera e del Clima, Consiglio Nazionale delle Ricerche, Roma 00133, Italy*

[3] *Istituto Nazionale di Oceanografia e di Geofisica Sperimentale, OGS, Trieste, 34010, Italy*

[4] *Stazione Zoologica Anton Dohrn, Villa Comunale, Napoli, 80121, Italy*

[5] *Department of Environmental Sciences, Informatics and Statistics, University of Venice, Mestre 30172, Italy*

[*]Corresponding Author. E-mail: ffalcini@isac.cnr.it

Abstract. We provide here supplementary materials such as methodologies, complementary data, and theoretical and experimental analysis. In particular, we provides general information about hydrologic features of the Ionian abyssal layer and the evolution of the Adriatic outflow characteristics.

Hydrographic conditions in the 2011

In situ measurements (Fig. S1) revealed a large inter-annual, thermohaline variability from 2003 to 2010 of the Adriatic outflow [1]. In 2003, the Adriatic Deep Water (AdDW) was not dense (i.e., cold) enough to reach the Ionian abyssal plain [2,3]. Then, it became colder until 2009, resulting in a large (negative) temperature difference, $\Delta T \sim -0.3°C$; then it warmed again starting from 2010.

Salinity, on the other hand, increased until 2007 and then reversed with a difference of about ΔS ~ -0.04 [1].

Temperature (*T*), salinity (*S*), and Dissolved Oxygen (*DO*) measurements carried out in the Ionian Sea during November 2011 (Fig. 3) show the presence of two Eastern Mediterranean Deep Water (EMDW) cores (*T* = 13.41-13.42 °C; *S* = 38.72-38.73). The first, located around 3000 m depth, is the "old EMDW" (*DO* = 4.3 ml/l); the second is "new EMDW" (*DO* = 4.4 ml/l) and it is observed in all stations that are below 3000 m depth (Fig. 3). This agrees with what observed in October 2009 and July 2010 over the same region [1]: the amount of Adriatic water (i.e., the strength of its mixing layer) that was produced during 2009-2010 did not cause an actual change of the thermoaline structure while differences in *DO* records, however, the continue advection of Adriatic water. Therefore, bottom waters in the Ionian Sea, observed in 2011, must be the result of the convolution of the non-stationary advection of the Adriatic mixing layer formed before 2007 [4], whose diffusion should act on a temporal scale between 3 and 7 years [5]. This proves that the stratification of the abyssal part of the Ionian Sea reflects the buoyancy flux variability from the Adriatic rather than local, external heat forcing that does not actually contribute to the bottom layer hydrography. Indeed, down-welling due to buoyancy loss depends not only on the amount heat sources acting at the sea surface (e.g., the wind stress) but also on mixed layer depth, stratification, and horizontal velocities [6]. Numerical simulations support this interpretation [7,8]: cold events (similar to the EMT) that produce large decadal variability of the deep water formation and show a hysteresis behavior, by activating the intrinsic nonlinearity of the Adriatic-Ionian-Aegean system. This results from the buoyancy transport at the source-water site of the Eastern Mediterranean [8] and agrees with what is generally found for the conveyor belt circulation of the Atlantic Ocean [9]. In addition, it is relevant to note the evolution of *T*-*S* diagrams (Fig. S3), which show two "elbows", not usually observed in the Mediterranean bottom layers in 2008 and 2011. The geometrical difference between the two "elbow" means more likely the transition from a thermocline to a halocline, and finally from the end of the halocline to the topographic bottom shows the evidence of a very deep mixed layer.

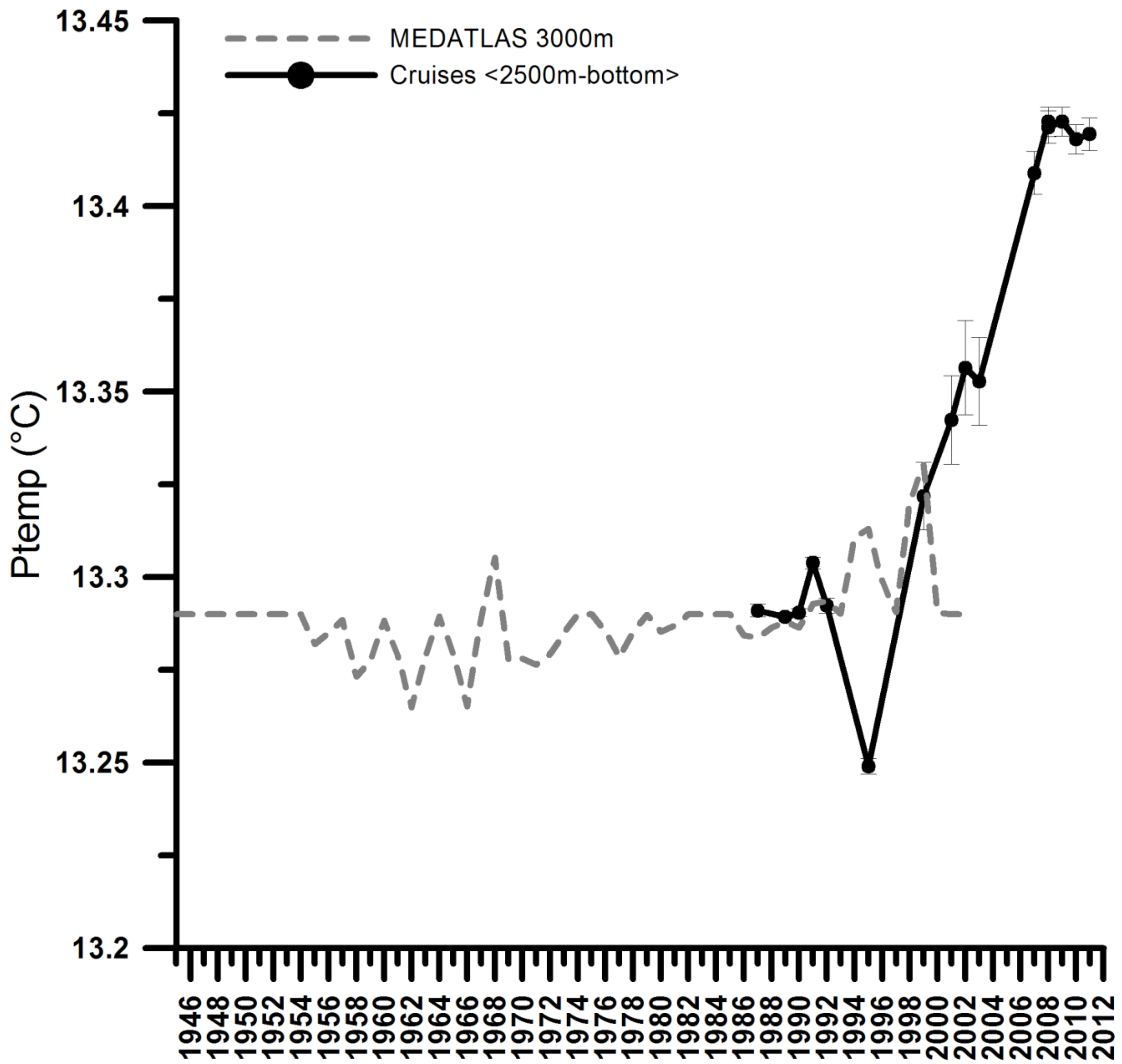

Figure S1. Time series of potential temperature (°C) in the central abyssal Ionian region. Grey dashed line refers to the reconstructed climatology (1945-2002) of ocean temperature, salinity, and density (MEDAR/MEDATLAS-II: Fichaut et al., 2003; Rixen et al., 2005). Black line/dots refer to *in-situ* data gathered through several oceanographic cruises: vertical CTD profiles are averaged in the layer 2500m-bottom (vertical bars show the standard deviation).

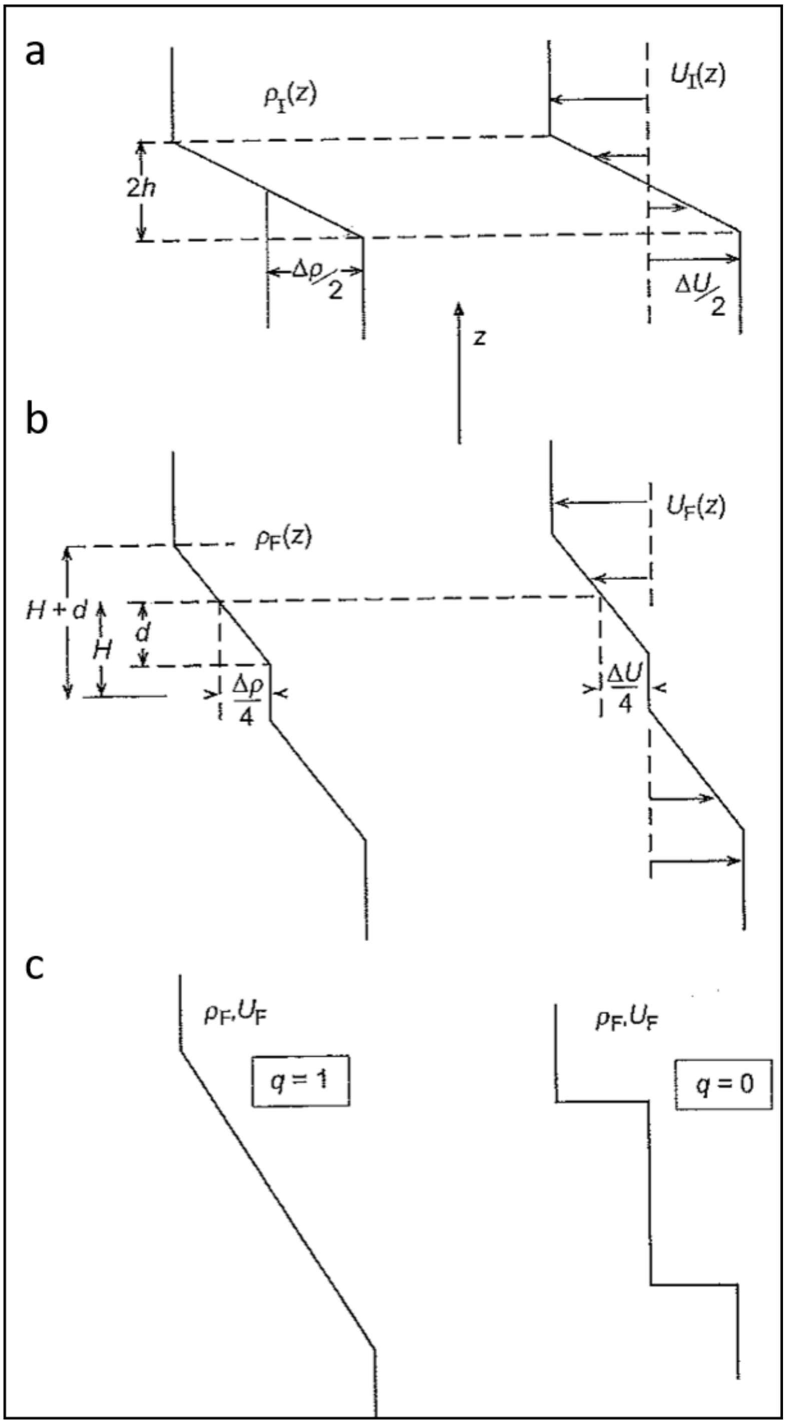

Figure S2. Mixing and step-like features. Sketch of the initial and final velocity and density before and after Kelvin-Helmholtz instability. From "The turbulent ocean" by S.A. Thorpe, 2007 (Figure 3.16, p. 107). Copyright 2005 by Cambridge University Press. Reprinted with permission. This figure cannot be reproduced, shared, altered, or exploited commercially in any way without the permission of Cambridge University Press, as it is copyrighted material and therefore not subject to the allowances permitted by a CC licence.

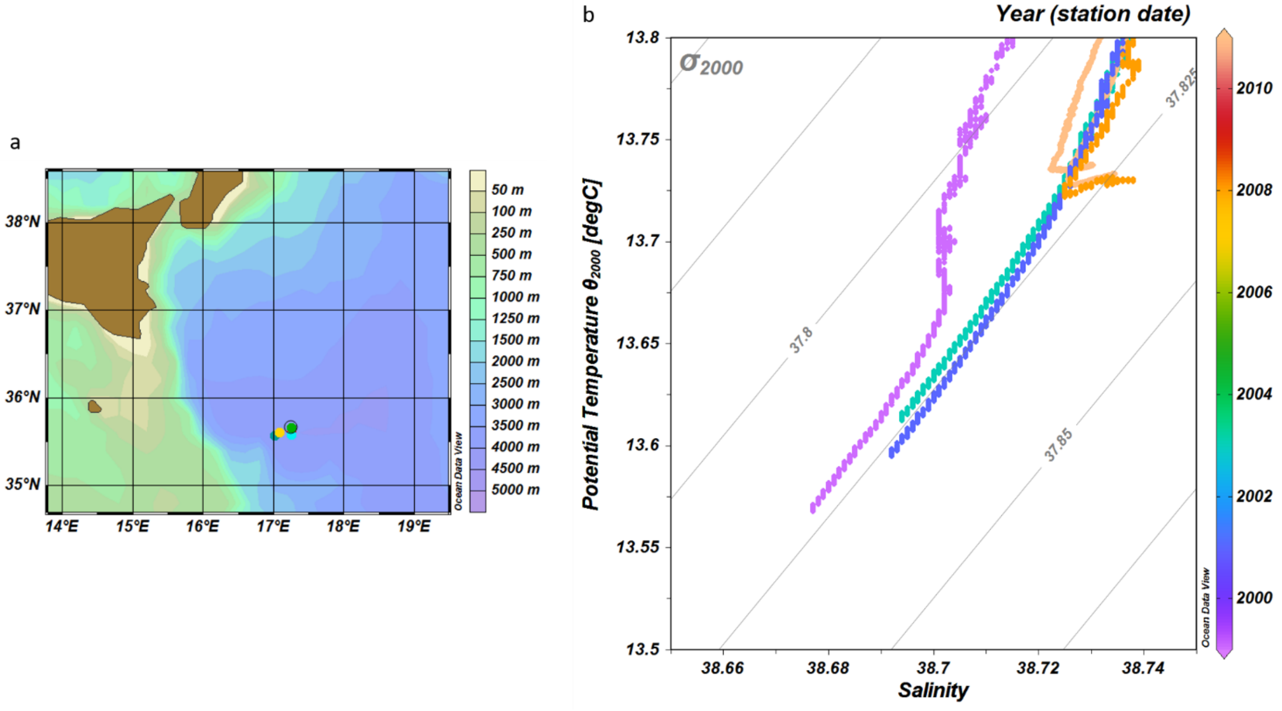

Figure S3. Mixing and "elbows" features. (a) geographic location of the deepest stations in the Ionian Sea. (b) T-S diagrams of the stations in panel (a), from 1999 to 2011. Figure created using Ocean Data View software (ODV - version, 4.7.4., Schlitzer, R., Ocean Data View, odv.awi.de, 2017).

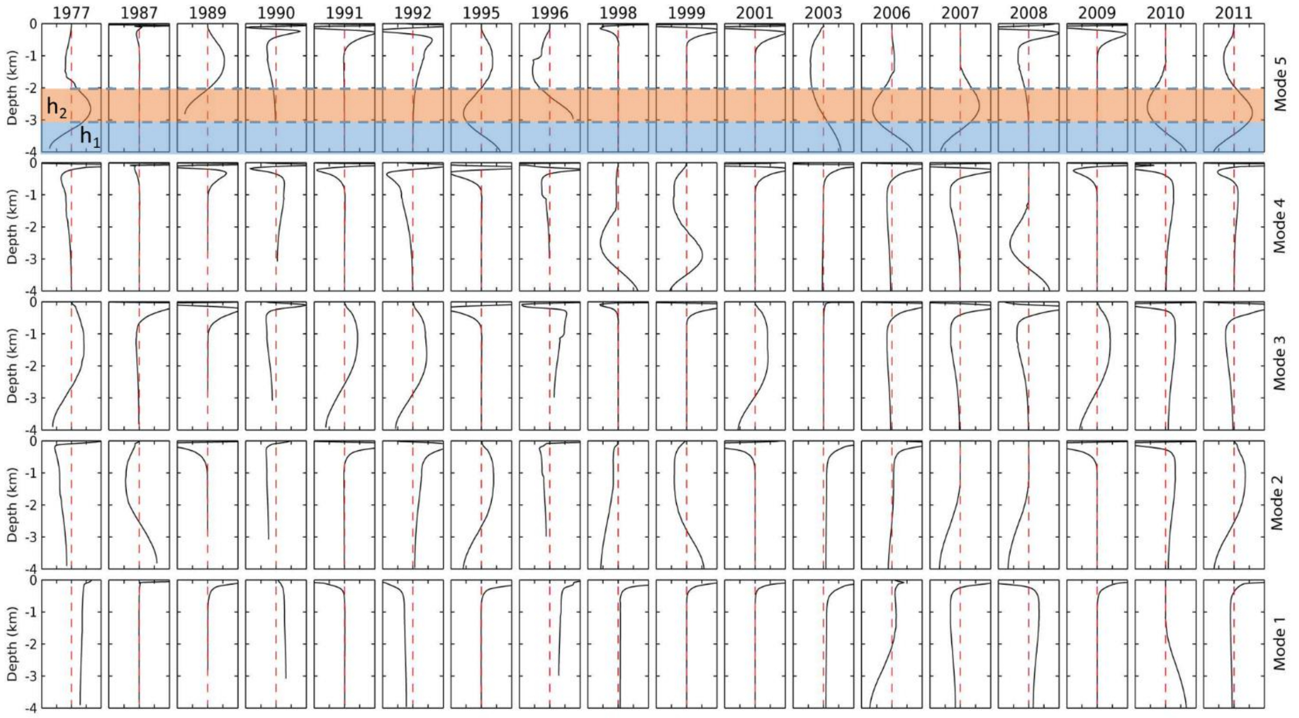

Figure S4. Vertical baroclinic modes. Temporal evolution of the vertical modes, from 1977 (left) to 2011 (right). For the 5th mode, we indicate the two baroclinic structures characterized by the equivalent depths (i.e., zero-crossing) h_1 and h_2, that were observed in 1977 and in 2011.

References

1. Bensi, M., Rubino, A., Cardin, V., Hainbucher, D. & Mancero-Mosquera, I. Structure and variability of the abyssal water masses in the Ionian Sea in the period 2003-2010. *J. Geophys. Res. Oceans* **118**, 931-943 (2013).
2. Hainbucher, D., Rubino, A. & Klein, B. Water mass characteristics in the deep layers of the western Ionian Basin observed during May 2003. *Geophys. Res. Lett.* **33**, (2006).
3. Rubino, A. & Hainbucher, D. A large abrupt change in the abyssal water masses of the eastern Mediterranean. *Geophys. Res. Lett.* **34** (2007).
4. Hainbucher, D., Rubino, A., Cardin, V., Tanhua, T., Schroeder, K. & Bensi, M. Hydrographic situation during cruise M84/3 and P414 (spring 2011) in the Mediterranean Sea. *Ocean Science*, **10**, 669 (2014).
5. Jungclaus, J. H. & Backhaus, J. O. Application of a transient reduced gravity plume model to the Denmark Strait Overflow. *J. Geophys. Res. Oceans* **99**, 12375-12396 (1994).
6. Spall, M. A. Buoyancy-forced downwelling in boundary currents. *J. Phys. Oceanogr.* **38**, 2704-2721 (2008).

7. Winton, M. & Sarachik, E. S. Thermohaline oscillations induced by strong steady salinity forcing of ocean general circulation models. *J. Phys. Oceanogr.* **23**, 1389-1410 (1993).
8. Amitai, Y., Ashkenazy, Y. & Gildor, H. Multiple equilibria and overturning variability of the Aegean-Adriatic Seas. *Glob. Planet. Change* **151**, 49-59 (2016).
9. Rahmstorf, S. On the freshwater forcing and transport of the Atlantic thermohaline circulation. *Clim. Dyn.* **12**, 799-811 (1996).
10. Fichaut, M., Garcia, M. J., Giorgetti, A., Iona, A., Kuznetsov, A., Rixen, M. & Group, M. MEDAR/MEDATLAS 2002: A Mediterranean and Black Sea database for operational oceanography. Elsevier Oceanography Series **69**, 645-648 (2003).
11. Rixen, M., Beckers, J. M., Levitus, S., Antonov, J., Boyer, T., Maillard, C., ... & Garcia, M. J. The Western Mediterranean Deep Water: a proxy for climate change. *Geophys. Res. Lett.* **32** (2005).
12. Thorpe, S. A. (2005). *The turbulent ocean*. Cambridge University Press.
